# Self-Collection Cervical Screening in the Asia-Pacific Region: A Scoping Review of Implementation Evidence

**DOI:** 10.1200/GO.22.00297

**Published:** 2023-02-01

**Authors:** Nicola Stephanie Creagh, Lucy Ann Patricia Boyd, Claire Bavor, Claire Zammit, Tessa Saunders, Anu Mary Oommen, Nicole Marion Rankin, Julia Mary Louise Brotherton, Claire Elizabeth Nightingale

**Affiliations:** ^1^ Centre for Health Policy, Melbourne School of Population and Global Health, The University of Melbourne, Melbourne, Victoria, Australia; ^2^ Community Health Department, Christian Medical College, Vellore, Tamil Nadu, India; ^3^ Australian Centre for the Prevention of Cervical Cancer, Carlton, Victoria, Australia

## Abstract

Although cervical cancer is a disease of inequity, it can be eliminated as a public health problem through vaccination, screening, and treatment. Human papillomavirus vaginal self-collection cervical screening is a high-performance test that can increase reach of screening. This review describes the different contexts and models of care used to pilot or implement self-collection within the Asia-Pacific, measures the extent that implementation outcome measures are reported and, where available, summarizes key implementation findings.

**METHODS:**

A scoping review was conducted by searching five databases of the peer-reviewed literature on June 20, 2022. Two researchers assessed eligibility and extracted data independently to the model of care used and the Conceptual Framework for Implementation Outcomes. A mixed-method consolidation of findings (quantitative: count and frequencies; qualitative: content analysis) was undertaken to narratively report findings.

**RESULTS:**

Fifty-seven articles, comprising 50 unique studies from 11 countries and two special autonomous regions, were included; 82% were conducted in trials. The implementation of self-collection was conducted in low- (2%), lower-middle– (32%), upper-middle– (32%), and high-income (35%) settings, with 10 different delivery models used; 80% delivered through practitioner-supported models with diversity in how samples were processed, and treatment was offered. Acceptability (73%) and appropriateness (64%) measures were most reported, followed by adoption (57%), feasibility (48%), and fidelity (38%). Only 7% of articles reported implementation cost or penetration measures. No articles reported sustainability measures.

**CONCLUSION:**

The literature confirms that self-collection cervical screening has been implemented within the Asia-Pacific region, with evidence demonstrating that it is acceptable and appropriate from the user's perspective. Well-designed, high-quality implementation trials and real-world evaluations of self-collection that report the breadth of implementation outcomes can support the progression toward the elimination of cervical cancer.

## INTRODUCTION

Cervical cancer is a disease of inequity that can be prevented. It is the fourth most common cancer diagnosed in women and people with a cervix (the term women is used hereafter, as included studies referred to women) and can result in undue suffering when diagnosed late.^1^ Worldwide, an estimated 604,000 new cases and 342,000 deaths occurred in 2020, mostly in low- and middle-income countries, with more than half in the Asia-Pacific region.^1,2^ Cervical cancer, in nearly all cases, is caused by persistent infection with oncogenic human papillomavirus (HPV).^3^ A combination of vaccination and early detection through screening can prevent cervical cancer.

**CONTEXT**

**Key Objective**
To our knowledge, we describe, for the first time, the different models of care used, and the implementation outcome evidence reported surrounding the implementation of human papillomavirus vaginal self-collection cervical screening in the Asia-Pacific region.
**Knowledge Generated**
Self-collection has been successfully implemented across the region, in low-, middle-, and high-income settings. The findings concur with global systematic reviews that self-collection is highly acceptable to women. Implementation outcome measures confirm that it is an appropriate and feasible screening option; however, evidence gaps remain, particularly for implementation cost/cost-effectiveness and long-term measures (penetration and sustainability).
**Relevance**
By harnessing the prevention tools available, including self-collection, cervical cancer can be eliminated as a public health problem in the region. Future trials and evaluations should consider the breadth of implementation outcome measures to ensure delivery models for self-collection suit the context, women's preference, and health system requirements, thus maximizing their sustainability and progression toward the elimination of cervical cancer.

In 2020, the WHO released a strategy to accelerate the global elimination of cervical cancer as a public health problem, defined as ≤ 4 cases per 100,000 women.^3^ To reach this goal, by 2030, countries need to achieve interim targets of 90% of girls fully vaccinated by age 15 years, 70% of women screening twice in their lifetime at 35 and 45 years with a high-performance test defined as a HPV DNA test,^4^ and 90% of women with pre- or invasive-cancer receiving treatment.^3^ Achieving these targets will avert 74.1 million cases and 62.6 million deaths by 2120.^5^

Historically, cervical screening has used cytology (Pap testing) at frequent intervals, requiring trained health professionals to use a speculum to collect cervical samples. The introduction of HPV DNA testing has changed the screening landscape and enabled the introduction of HPV vaginal self-collection (hereafter self-collection). Self-collection, where a woman collects her own vaginal sample, has demonstrated efficacy for the detection of underlying cervical high-grade lesions.^6^ For women, it can overcome personal barriers related to the invasiveness of clinician-collected screening and can increase participation in screening among underscreened and underserved populations, including among First Nations women in countries with a history of colonization.^6-11^ Removing the need for trained health care providers to perform the testing also creates opportunities for flexible delivery models that can be tailored to local contexts and health care systems, thus improving accessibility and equity to screening.^6,8,12^

Global efforts to pilot or implement self-collection are gaining momentum. A recent review identified that globally, 35% of countries with an established HPV-based screening program have implemented self-collection as a primary or additional screening modality, with 10 countries undertaking pilot studies before implementation.^13^ In 2019, however, the WHO country summaries indicated that in the Western Pacific region (WPRO) and the Southeast Asian region (SEARO), only 48% and 64% of countries (respectively) had an organized screening program.^14,15^ Likewise, only 11% of countries in WPRO and no countries in SEARO had implemented HPV testing.^14,15^ Screening coverage was also low, with only 4% and 18% of WPRO and SEARO countries, respectively, having achieved ever in lifetime screening coverage of > 70%,^14,15^ meaning there is significant work required to reach the elimination interim screening target for the regions. Self-collection could be a highly useful tool in the region's progression toward this target.

This scoping review aims to understand the current implementation evidence for self-collection in the Asia-Pacific region by (1) describing the different contexts and models of care used to pilot or implement self-collection, (2) mapping the evidence within the peer-reviewed literature to the implementation outcomes framework by Proctor et al,^16^ and (3) summarizing implementation evidence to identify areas for future research to inform policy and practice.

## METHODS

The scoping review protocol was registered and published on the Open Science Framework website^17^ and conducted in accordance with the scoping review framework by Levac et al^18^. The review is reported following the PRISMA Extension for Scoping reviews reporting checklist^19^ (Data Supplement).

### Search Strategy

Database searches were conducted on MEDLINE (Ovid), Scopus, CINAHL, Web of Science, and PubMed using the terms: cervical and human papillomavirus, self-collection, and implementation outcome terms (full search strategy—Data Supplement). Search limitations included peer-reviewed articles published in English from inception until June 20, 2022 (search date). The reference lists of included studies were assessed for additional articles.

### Article Selection Criteria

Study designs that were experimental, observational, quantitative or qualitative, and presented one or more implementation outcomes relating to self-collection in a trial or real-world setting were included. Studies were excluded if they discussed self-collection in a theoretical context (in the absence of a trial or implementation). The exclusion criteria after full-text review were later refined to limit inclusion to studies within the Asia-Pacific region, defined as countries within the WPRO and SEARO WHO regions. Table 1 presents an overview of the implementation outcomes, their definitions, and operationalization in the context of this review, using the outcomes for implementation research framework by Proctor et al.^16^

**TABLE 1:**
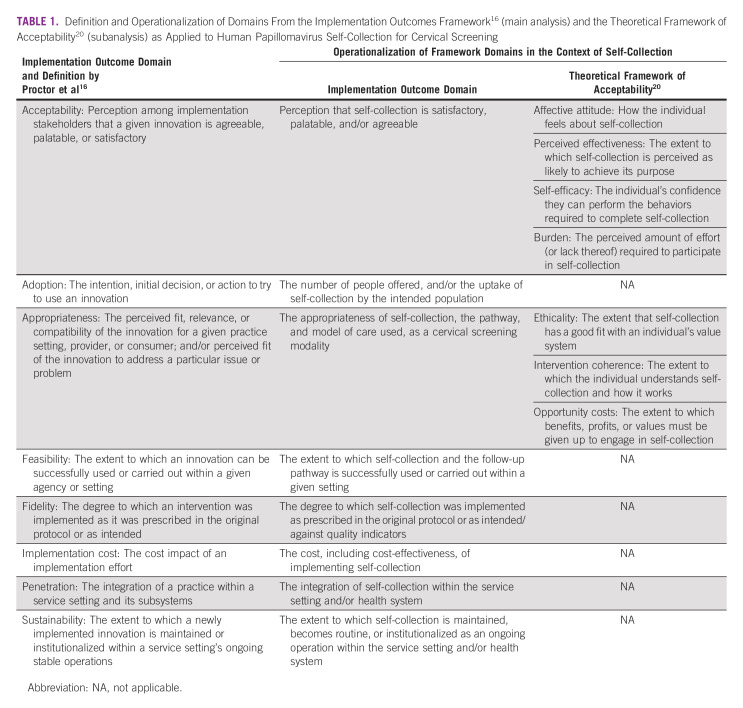
Definition and Operationalization of Domains From the Implementation Outcomes Framework^16^ (main analysis) and the Theoretical Framework of Acceptability^20^ (subanalysis) as Applied to Human Papillomavirus Self-Collection for Cervical Screening

### Article Selection

All relevant citations were imported into Covidence systematic review software (Veritas Health Innovation, Melbourne, Australia). After duplicates were removed, titles and abstracts were screened by two independent researchers from a group of six authors (N.S.C., L.A.P.B., C.B., C.Z., T.S., and A.M.O.). Articles then underwent full-text review by two independent researchers from a group of six authors (N.S.C., L.A.P.B., C.B., C.Z., T.S., and A.M.O.) to determine eligibility for inclusion. At both stages, conflicts were assessed by a third researcher (N.S.C. and C.E.N.) where required.

### Data Extraction

Covidence was used to extract the following data items from included articles: country, self-collection model of care, HPV swab and assay, research design, type of follow-up/treatment provided, and implementation outcome measures. Two researchers from the team of seven authors (N.S.C., L.A.P.B., C.B., C.Z., T.S., A.M.O., and C.E.N.) independently extracted data, with any conflicts resolved through discussion by researchers who extracted data from each article.

### Data Synthesis

Extracted data were exported into Excel and synthesized using a mixed-methods approach. For quantitative measures, counts and frequencies were computed to describe the frequency of variables, such as the country, model of care, and implementation outcome measures (Table 1). The extracted implementation outcome measures data were summarized if required or kept verbatim and imported into NVivo (release 1.6.2.; QSR International Pty Ltd, Melbourne, Australia) for a qualitative content analysis, which adhered to the process outlined by Forman and Damschroder.^22^ This involved becoming familiar with the data within each implementation outcome, developing a coding scheme (final coding framework: Data Supplement), and arranging the data. During extraction, we identified an overlap in definitions and measures for acceptability and appropriateness. We therefore conducted a subanalysis of these domains using the Theoretical Framework for Acceptability,^20^ which ensured that the coding scheme and thus, interpretation of the data was framework-informed. The qualitative content analysis was conducted by one researcher (N.S.C.) and confirmed by another (C.B.; Data Supplement).

## RESULTS

**FIG 1:**
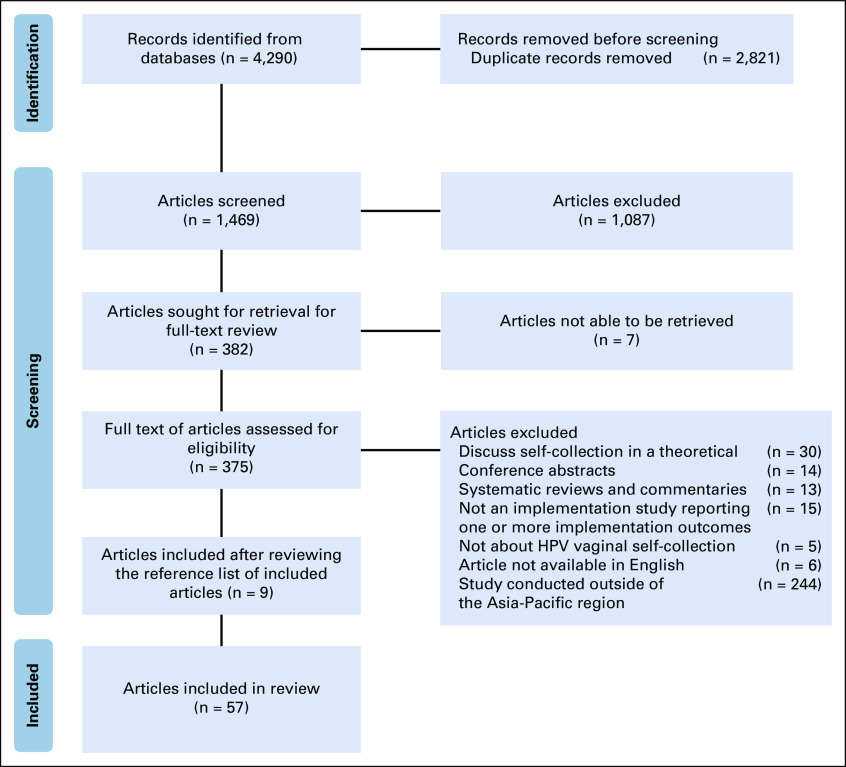
Identification and selection of articles for the scoping review. HPV, human papillomavirus.

The search strategy produced 1,469 articles (2,821 duplicate articles removed; Fig 1). After title and abstract screening, 382 full-text articles were reviewed, 90 of which were excluded on the basis of the original inclusion criteria. A further 244 articles were excluded that were not from the Asia-Pacific region.

A total of 57 articles were included, with nine added after reviewing the reference list of included articles. These 57 articles reported on 50 unique studies conducted in 11 countries and two special administrative regions (Taiwan and Hong Kong; Table 2, data chart in the Data Supplement). Of the articles included in this review, one (2%) was conducted in a low-income country, 18 (32%) in lower-middle–income countries, 18 (32%) in upper-middle–income counties, and 20 (35%) in high-income countries (Table 3). Most (n = 41; 82%) studies implemented self-collection in a trial setting (where self-collection is only available within a research program), with nine (18%) studies conducted in the context of real-world program implementation.

**TABLE 2:**
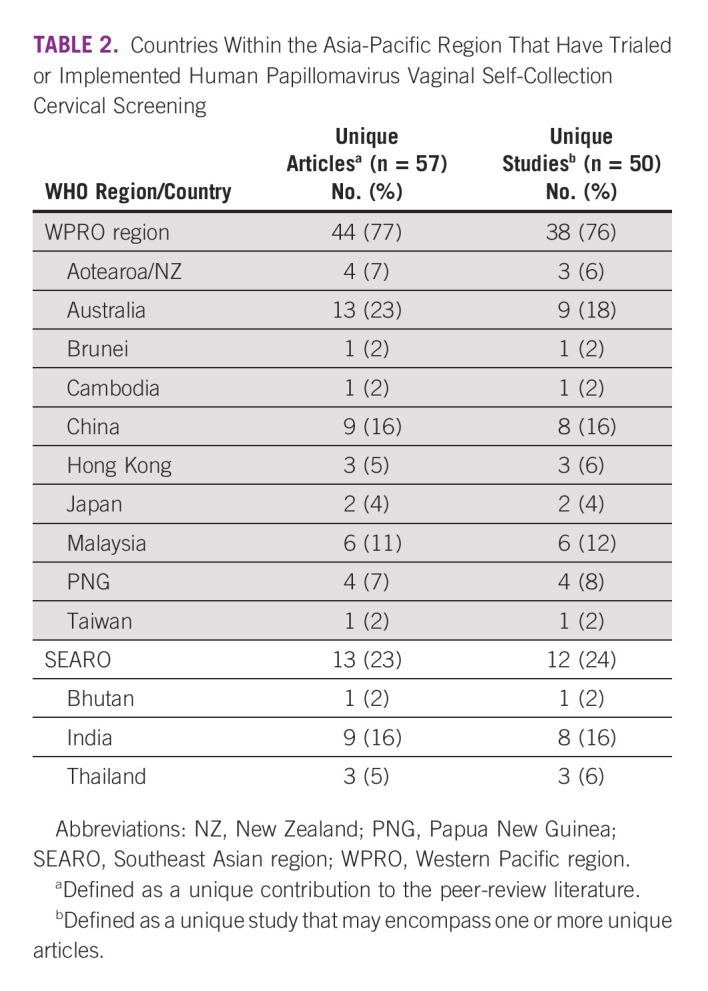
Countries Within the Asia-Pacific Region That Have Trialed or Implemented Human Papillomavirus Vaginal Self-Collection Cervical Screening

**TABLE 3:**
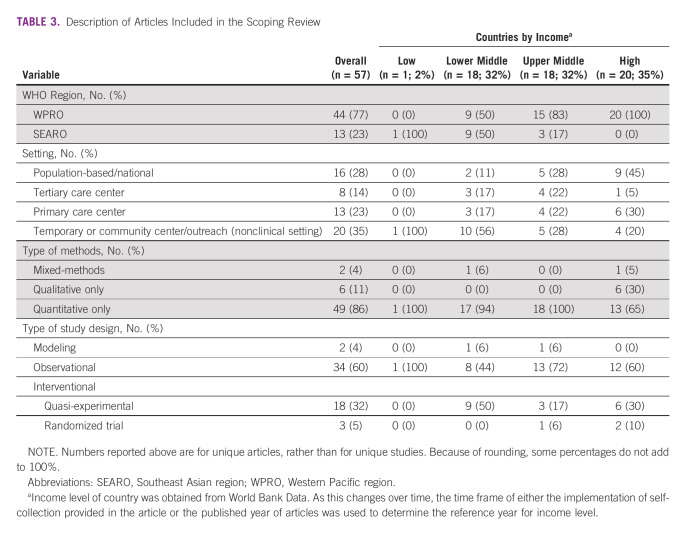
Description of Articles Included in the Scoping Review

### Model of Care

Self-collection was provided through 10 broad models of care, in different combinations of (1) methods of providing the collection device (practitioner-supported, door-to-door, opt-in or opt-out mail-out), (2) test strategies (laboratory-based or point-of-care), and (3) treatment strategies (referral or screen-and-treat); one article from Taiwan provided no description^23^ (Table 4).

**TABLE 4:**
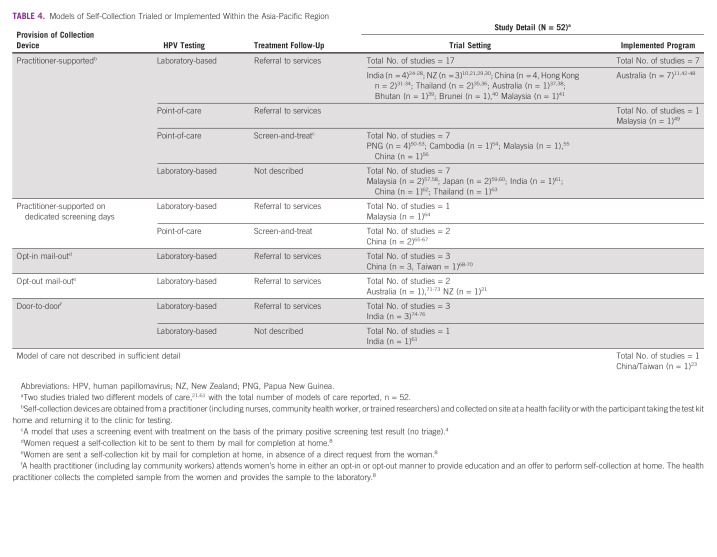
Models of Self-Collection Trialed or Implemented Within the Asia-Pacific Region

Of the 52 models or care trialed or implemented, most were practitioner-supported (n = 42; 81%), four used door-to-door models (n = 4; 8%), three used opt-in (n = 3, 6%), and two used opt-out (n = 2; 4%) mail approaches. All door-to-door models were implemented in India (a low-middle–income country), with practitioner-supported and mail-out models implemented in settings of various income levels. Most studies (n = 41; 79%) transported samples to a laboratory for testing, with 10 (19%) using point-of-care testing devices. Follow-up testing/treatment was mostly provided through referrals (n = 34; 65%), nine studies (17%) conducted in lower-middle– or upper-middle–income level countries used screen-and-treat models and nine studies (17%) not describing follow-up pathways (Table 4).

### Implementation Outcome Measures

**FIG 2:**
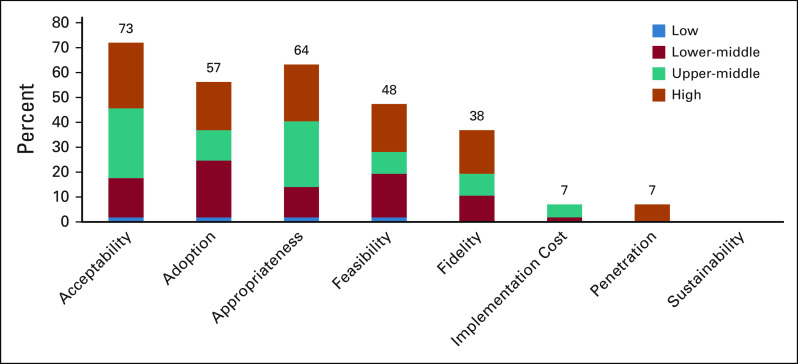
The reporting of implementation outcome measures surrounding human papillomavirus vaginal self-collection in the Asia-Pacific region on the basis of the framework by Proctor et al (articles, n = 57)^16^. The figure legend refers to income level of countries, obtained from the World Bank. As this changes over time, the time frame of either the implementation of self-collection provided in the article or the published year of articles was used to determine the reference year for income level.

Figure 2 presents the number of articles reporting each implementation outcome. This evidence is summarized by each implementation outcome domain below.

#### Acceptability.

A total of 40 studies (41 articles, 73%; 2% low-, 22% lower-middle–, 39% upper-middle–, and 37% high-income countries) reported a range of measures that reflected good acceptability of self-collection.^10,11,23-27,29-37,39-47,49,50,54,57-60,63-65,68-71,74^ Studies reported high satisfaction of self-collection among women,^10,11,30,36,42,43,50,58,62,63,65,68,70^ that women were willing to perform self-collection again,^10,11,23,27,29-31,35,41,49,57,58,60,68,69^ and to recommend self-collection to others.^10,11,27,29,30,32,49,50,68^ Self-collection was commonly described as easy,^10,11,26,29,30,33-36,39-41,43,50,57-60,64,69,71^ comfortable or convenient,^10,11,27,29-33,35,36,41,43,49,57,58,63,65,68-71,74^ less embarrassing,^10,11,27,29-32,36,57-60,62,63,65,69-71^ and less painful^10,11,27,29,30,32,34,36,39,41,43,57-60,63,70,71,74^ than clinician-collected screening.

Of the studies that reported women's confidence in performing self-collection, 10 (67%) reported a high level of confidence,^30,32,40,41,43,54,58,62,65,71^ whereas five reported lower self-efficacy among participants.^10,31,37,64,70^ In one Australian study, women were concerned about correctly performing self-collect before the test, but noted concerns were alleviated after performing it.^43^

Five studies, all conducted in Australia (high-income setting), considered the acceptability of self-collection from the practitioner's perspective.^42,44-47^ Many practitioners were supportive of the inclusion of self-collection within the national program.^42,44,45,47^

#### Adoption.

A total of 27 studies (57%, 32 articles; 3% low-, 41% lower-middle–, 22% upper-middle–, and 34% high-income countries), 26 of which were trials, reported adoption measures.^10,11,21,24-26,30,32,33,35,37-41,50-52,56,57,59,61,62,68-76^ High rates of uptake were reported for models implemented in low- or low-middle–income level settings that used point-of-care testing (n = 4; 68%-100%)^50-52,56^ and door-to-door models (n = 3; 81%-97%).^74-76^ Two studies in Papua New Guinea, which used a point-of-care screen-and-treat model, reported that 100% of invited participants undertook self-collection.^50,51^ Sixteen (94%) studies using a practitioner-supported model reported uptake rates of > 50%,^10,11,25,26,30,32,37-40,57,59^ with five studying conducted in lower-middle– or upper-middle–income level settings reporting uptake > 90%.^24,33,35,41,62^ A New Zealand study that used a practitioner-supported model to engage specific underscreened cultural groups reported an uptake of 24%.^30^

Most studies investigating opt-in mail-out models of care (n = 3; upper-middle– income level settings)^68-70^ reported higher uptake compared to opt-out models of care (n = 1 study reported in three articles, high-income level setting; 61.1%-73% *v* 9.1%-20.3%).^71-73^ However, one study conducted in Taiwan, using an opt-in mail-out model, reported an uptake of only 2.6% (n = 282/10,693 invited to opt-in).^68^ Two studies compared practitioner-supported models with in-clinic collection versus mail-out or at-home collection models (n = 1 at-home testing, n = 1 opt-out); higher uptake was reported in mail-out/at-home testing models.^21,27^ We note that in models that use a population-based mail-out approach, it is not always possible to confirm receipt of test, nor eligibility for screening (ie, hysterectomy). Thus, these lower-adoption measures may not accurately reflect participant engagement in programs.

Two studies reported lower uptake among women age > 45 years,^39,61^ but this was not found in a third study.^70^ A study conducted in Bhutan reported higher uptake by participants from a rural area compared with metropolitan locations (70% *v* 33%).^39^ A study in New Zealand reported higher uptake among Māori and Asian women compared with Pacific women.^65^

#### Appropriateness.

A total of 34 studies (36 articles, 64%; 3% low-, 19% lower-middle–, 42% upper-middle–, and 35% high-income countries) reported appropriateness implementation measures.^10,11,23,26,27,29-33,35-37,39-44,47,49,50,56-60,62-65,68-71,74^ Of the studies that reported preference between self-collected or practitioner-collected screening, most (n = 22/24) reported women's preference for self-collection.^10,26,27,29-33,35,36,43,49,57-60,63,65,69,70,74^ Studies reported that self-collection was empowering and provided bodily autonomy,^10,30,43^ and was culturally appropriate for specific population groups.^30,35,47,63^ One Australian study reported that practitioners viewed self-collection as appropriate to engage underscreened or never-screened individuals, but not for all screeners.^47^

Studies in Brunei (n = 1) and Malaysia (n = 1) reported that most women (54.6%-60%) preferred practitioner-collected screening.^40,64^ Common concerns among women and practitioners around self-collection were its accuracy compared with practitioner-collected screening^29-31,36,42-44,59,63,65^ and inadequate collection by women.^11,29,41,47,62-65^

Eight studies, seven of which investigated practitioner-supported models, reported participant's preference for location of testing.^30,33,35,36,41,58,62,71^ Six of the eight studies (75%) reported a higher preference for at-home testing^30,33,36,58,62,71^ compared with a health care setting.^35,41^ Five studies reported that most women found the provided pictorial and/or verbal instructions appropriate.^11,40,60,63,71^ Three studies reported on the appropriateness of the cost of the kit to screening participants.^23,36,41^ In two studies, most women were willing to purchase the self-collect test if cost was low,^36,41^ whereas one study reported that when women's priority consideration was cost, and they were least likely to undertake self-collection.^23^

#### Feasibility.

Feasibility implementation measures were reported by 25 studies (27 articles, 48%; 4% low-, 37% lower-middle–, 19% upper-middle–, and 41% high-income countries), most (n = 22) within a trial setting.^10,11,21,24,26,28,30,34,37-40,42,49,50,52,54,56,66-69,76^

Adherence to follow-up ranged from 29.8%-100%.^10,11,21,26,28,30,34,37-40,48-50,52,54,56,66-69,72,73,76^ Studies from Malaysia (n = 1), Bhutan (n = 1), Papua New Guinea (n = 2), Brunei (n = 1), China (n = 1), New Zealand (n = 2), and Australia (n = 2) reported adherence to a specific pathway of follow-up of > 90%.^21,30,34,37,39,40,49,50,52,73^ Three studies, one of which conducted in a program setting, reported follow-up rates of < 60%.^11,68,73^ One study from Australia, implementing a practitioner-supported model of care, found lower follow-up rates among participants testing HPV+ non-16/18, compared with women testing HPV+ 16/18.^11^ Another Australian trial with an opt-out mail-out model reported lower follow-up among women who were provided a second self-collection kit for a repeat HPV test at 12 months, compared with the first round of screening.^73^ A study from China reported that 29.8% of HPV+ women accessed follow-up within the study timeframe, but registry data suggested that most (70%) received follow-up beyond the study.^68^

Absence of symptoms was reported as negatively influencing adherence to follow-up in three studies,^24,29,56^ and support from practitioners, friends, or family was found to positively influence attendance for follow-up in two studies.^10,42^

Time to follow-up was reported in two studies, with one reporting that the median time to follow-up was 3 months for women requiring colposcopy/histology and 1.1 months for women requiring cytology^73^ The other study reported that all participants completed follow-up between 2 weeks to 6 months.^30^ The time implications for health services and practitioners were reported in two studies. In Papua New Guinea, it was reported that HPV+ women required longer visits than HPV-negative women within a point-of-care screen-and-treat model.^50^ In New Zealand, a study focused on engaging underscreened Māori, Pacific, and Asian women reported that providing follow-up care to women was time-intensive, requiring approximately 5 hours of skilled nursing time per patient.^30^

#### Fidelity.

Fidelity implementation measures were reported by 19 studies (21 articles, 38%; 29% lower-middle–, 24% upper-middle–, and 48% high-income level setting).^11,21,28-30,34,35,37-39,42,48,49,51,56,61,63,69,72,73,76^ Of the 15 studies that reported rates of invalid samples (13 within trials), 10 studies conducted in lower-middle–, upper-middle–, and high-income level settings reported that < 4% of test results were invalid,^11,21,28,37-39,48,49,61,63,72,73,76^ with two studies conducted in China (an upper-middle–income country) and Papua New Guinea (a lower-middle–income country) reporting that all samples were satisfactory for testing.^34,51^ One small New Zealand study comparing swab types reported an invalid test rate of 8.6%, all likely because of the preanalytic handling of that brand of swab (n = 3/35).^29^ A larger study conducted in Thailand reported that 25% of samples tested were inconclusive, with no reason provided (n = 67/267).^35^ Both studies with high invalid rates used a practitioner-supported model of care and tested samples by polymerase chain reaction–based assays. Another study reported high wastage rates with invalid runs on the signal amplification assay careHPV but did not report invalid rates.^39^ One study with a low invalid rate of 2.5% (n = 2/79) reported that no cell content was detected in those samples and suggested that these women returned the self-collection kit without having performed the test.^37,38^

Of the studies that reported protocol variations, all but one were conducted in trial settings.^11,30,37-39^ Protocol variations reported included expanding the study inclusion criteria to account for difficulties in recruiting participants,^30^ extending time for data collection because of difficulties retaining community engagement workers and extending time to provide results because of nonattendance,^30^ changes to the way results were delivered,^11^ and modifications to the follow-up pathway to account for the high emotional stress of participants following a HPV+ result.^37,38^ Outside trial settings, an Australian study evaluating the introduction of self-collection found there were varied interpretations and applications of the guidelines by practitioners.^42^

#### Implementation cost.

Four studies (four articles, 7%; 25% lower-middle– and 75% upper-middle–income countries) reported cost measures.^32,53,55,68^ Two studies from China reported real costs related to the implementation of a pilot program. One study implemented a practitioner-supported model of care and reported the cost of processing each self-collected sample as $16.30 US dollars (USD), with the cost increase per HPV+ case being $86.10 USD.^32^ In this context, self-collection compared favorably with cytology, which was estimated to cost $99.00-$297.00 USD before referral to colposcopy.^32^ A second paper trialing an opt-in mail-out model reported the overall trial cost and the cost per cervical intraepithelial neoplasia 2+ case detected, but no assessment was provided on cost-effectiveness.^68^ Two economic modeling studies, which considered projected costs for Papua New Guinea and Malaysia, concluded that cervical screening using a point-of-care screen-and-treat model with self-collection was cost-effective in these settings.^53,55^ In Malaysia, modeling demonstrated that cost-effectiveness is dependent on high rates of follow-up.^55^ In Papua New Guinea, compared with HPV screening, primary screening using Visual Inspection with Acetic Acid was not considered cost-effective even if Visual Inspection with Acetic Acid achieved a sensitivity of 70%.^53^

#### Penetration and sustainability.

No studies reported on sustainability. Four studies (four articles, 7%), all conducted in Australia (a high-income country), which used a practitioner-supported model of care, reported on penetration measures reflecting the first 2 years after the introduction of self-collection.^42,44,47,48^ One study, using observational data from the national registry, reported that HPV tests on a self-collection sample were conducted for only 0.1% of women attending screening.^48^

Three studies were qualitative evaluations reporting on practitioner's understanding and utilization of self-collection,^42,44,47^ and one study reported on women's understanding and use of self-collection.^42^ Awareness of self-collection among women (who had undertaken self-collection) was reported as minimal until their practitioner offered the test.^42^ Practitioners reported mixed experiences about their awareness of the availability of self-collection. Some found out about its availability 12 months after its introduction^42^ and most general practitioners had limited experience with self-collection, having not yet integrated it into their clinical practice.^47^ One study found that, although practitioners had been communicating about self-collection to their patients, challenges with program implementation restricted their capacity to provide self-collection as a routine part of their care.^44^

## DISCUSSION

To our knowledge, this paper represents the first consolidation of evidence for the implementation of self-collection cervical screening across the Asia-Pacific region, which is a highly diverse region comprising low-, lower-middle–, upper-middle–, and high-income countries, with considerable variation in health care systems structures. Reviewing data against the implementation outcomes framework by Proctor et al (2011)^16^ indicates that self-collection is feasible to implement in low-, middle-, and high-income level settings, and is highly acceptable for women, which concurs with global reviews surrounding the acceptability of self-collection.^9,77^ Application of this framework also highlighted gaps in the implementation evidence for the region.

This review highlights that the most trialed or implemented model of care in the region is practitioner-supported models, used in settings of various income-level classifications. Existing global systematic reviews have demonstrated the effectiveness of self-collection for mail-out and door-to-door models in increasing participation in screening^6,8^; however, this strong evidence is yet to be generated for practitioner-supported models. Furthermore, very few studies have assessed the implementation of self-collection from a practitioner's perspective. Practitioner-supported models have unique considerations in terms of their workforce and infrastructure requirements, which may compromise their scale-up and sustainability of implementation, particularly for low-resource settings. Although they may be the most appropriate model in many settings, further research should consider cost-effective analyses, structures required to implement and maintain the delivery of self-collection within a health service and system, and practitioners' perspective of implementation.

Most studies in this review used centralized laboratory testing and referrals for follow-up and treatment, which require substantial infrastructure. Two studies reported high invalid rates of testing. Although clinical trials report low rates of invalids when using polymerase chain reaction–based assays,^78^ this finding demonstrates the importance of measuring fidelity to testing protocols as unsatisfactory rates can be affected by other factors including the preanalytic handling of the swab, presence of lubricants, or a lack of endogenous material, potentially because participants may agree but not undertake the test.^12,38^ This review found that adherence to follow-up was reasonable, with some studies achieving > 90%. Triage and treatment algorithms used within studies were complex, which may rely on various health system factors to support follow-up adherence. This warrants further investigation to determine enabling factors in each setting. In instances where high rates of follow-up were achieved, understanding the facilitators and system-level factors that supported this is imperative to inform delivery in other settings. Articles included in this review, however, provided limited insights around factors that support adherence beyond emotional support provided by practitioners, friends, or family. It is of critical importance that models are implemented that support those least served to engage in follow-up and treatment to ensure programs equitable improve outcomes for all. Health-system initiatives such as patient navigators and high-quality information systems that provide recall services to support adherence to follow-up and record ethnicity indicators are among some initiatives that can promote equity within programs.^79,80^ This is particularly important, given that access to treatment of precancer and cervical cancer is an essential component of the global elimination strategy.

Emerging evidence from the region highlights that screen-and-treat models, with point-of-care testing, are feasible to implement within low-resource settings.^50-52^ Screen-and-treat models are endorsed by the WHO and are likely essential to enable countries with a lower level of resources in the region achieve the elimination targets for screening and precancer treatment.^4^ All studies that explored a screen-and-treat model were trials, and although data indicate these models are acceptable, appropriate, feasible, and likely cost-effective, there were no studies describing this model in a sustainably implemented program. Thus, penetration and sustainability outcomes require ongoing evaluation.

Adoption was predominately measured in trials, which were likely well resourced to support implementation, and therefore cannot necessarily inform the uptake of self-collection within programs. Cost-effectiveness data specific to the region are limited, and the only data on penetration of self-collection are from a high-resource setting (Australia), where self-collection has been available in a restricted way within the national program for nearly five years and recently made available as a choice for all people undergoing screening.^42,43^ No studies investigated the sustainability of self-collection. This is likely because of most studies being conducted in trial settings and because self-collection and HPV screening remain relatively new, with programs yet to reach a second round of HPV screening.

The limitations of this scoping review include that it was restricted to peer-reviewed articles published in English, which may have limited our ability to accurately represent the extent to which self-collection has been trialed or implemented within the Asia-Pacific region. Furthermore, no risk of bias was performed as this is beyond the scope of a scoping review. By contrast, a strength of this review is that we have documented implementation outcome measures that have been reported for the region for the first time, to our knowledge, in a comprehensive way.

Cervical cancer is a disease of inequity, but by harnessing available testing strategies, including self-collection, this unnecessary burden can be alleviated. Self-collection can support progression toward the WHO goal to eliminate cervical cancer as a public health problem by 2,120 and progress toward achieving the sustainable development goals of gender equality, universal health care, and reduced inequalities.^81^ This review concurs with systematic review evidence that self-collection is acceptable to the women in the Asia Pacific region.^9,77^ In addition, the evidence consolidated presented sheds light on the high levels of adoption, appropriateness, and feasibility of self-collection in the region. However, gaps in the evidence remain, relating to long-term implementation outcomes, and the trial nature of many studies may limit the applicability of findings for national and regional programs. As further unique models of care are trialed and more programs are implemented, well-designed, high-quality implementation trials and real-world evaluations that consider the breadth of implementation outcomes (including the cost, penetration, and sustainability of self-collection) will be needed, to understand how delivery models can be adapted to suit the local context, women's preferences, and the health-system requirements of diverse settings within the region.
